# *Drosophila para*^*bss*^ Flies as a Screening Model for Traditional Medicine: Anticonvulsant Effects of *Annona senegalensis*

**DOI:** 10.3389/fneur.2020.606919

**Published:** 2021-01-13

**Authors:** Samuel S. Dare, Emiliano Merlo, Jesus Rodriguez Curt, Peter E. Ekanem, Nan Hu, Jimena Berni

**Affiliations:** ^1^School of Medicine, Kabale University, Kabale, Uganda; ^2^Department of Anatomy, Kampala International University, Kampala, Uganda; ^3^Facultad de Medicina, Instituto de Fisiología y Biofísica (IFIBIO)-Houssay, Universidad de Buenos Aires - Consejo Nacional de Investigaciones Científicas y Técnicas (CONICET), Buenos Aires, Argentina; ^4^School of Psychology, University of Sussex, Brighton, United Kingdom; ^5^Department of Zoology, University of Cambridge, Cambridge, United Kingdom; ^6^Brighton and Sussex Medical School, University of Sussex, Brighton, United Kingdom; ^7^Anatomy Unit, Institute of Biomedical Sciences, College of Health Sciences, Mekelle University, Mekelle, Ethiopia

**Keywords:** *Annona senegalensis*, bang sensitive, *Drosophila melanogaster*, epilepsy, *para*^*bss*^, phenytoin, seizure, eas^2*F*^

## Abstract

Epilepsy is among the most common serious neurological disorders and affects around 50 million people worldwide, 80% of which live in developing countries. Despite the introduction of several new Anti-Epileptic Drugs (AEDs) in the last two decades, one third of treated patients have seizures refractory to pharmacotherapy. This highlights the need to develop new treatments with drugs targeting alternative seizure-induction mechanisms. Traditional medicine (TM) is used for the treatment of epilepsy in many developing countries and could constitute an affordable and accessible alternative to AEDs, but a lack of pre-clinical and clinical testing has so far prevented its wider acceptance worldwide. In this study we used *Drosophila melanogaster paralytic* ^*bangsensitive*^ *(para*^*bss*^*)* mutants as a model for epileptic seizure screening and tested, for the first time, the anti-seizure effect of a non-commercial AED. We evaluated the effect of the African custard-apple, *Annona senegalensis*, which is commonly used as a TM for the treatment of epilepsy in rural Africa, and compared it with the classical AED phenytoin. Our results showed that a stem bark extract from *A. senegalensis* was significantly more effective than a leaf extract and similar to phenytoin in the prevention and control of seizure-like behavior. These results support that *Drosophila* constitutes a robust animal model for the screening of TM with potential value for the treatment of intractable epilepsy.

## Introduction

Epilepsy is among the most common serious neurological disorders and affects around 50 million people worldwide, with 80% of them living in developing countries (1–4). Epilepsy can cause frequent seizures, which are brief episodes of involuntary shaking involving part of or the entire body, and are sometimes accompanied by a loss of consciousness. Seizures can vary in intensity from brief lapses of attention or muscle jerks, to severe and prolonged convulsions. They can also vary in frequency, from less than one per year to several per day.

These episodes are triggered by hyper-excitation and/or abnormal synchronization of activity across neuronal circuits (5). The causes of epilepsy are attributed to acquired vs. genetic factors. Acquired epilepsy, such as those resulting from trauma, stroke, neoplasm, infection, congenital malformations, birth anoxia and autoimmune, represent slightly more than a quarter of the cases. Epilepsy with complex inheritance, ranging from mono-gene mutations to the presence of modifiers and susceptibility alleles has emerged as the main causes of idiopathic epilepsy (6). Mutations in channels, that determine cellular excitability, have been associated with a wide range of epilepsies (5, 7). Amongst them is the sodium channel SNC1A, the human ortholog of the *para*^*bss*^ gene of *Drosophila melanogaster*, with over 600 different mutations found in patients (8). Links to other ionic channels (e.g., voltage-gated sodium channel a2 gene subunit SCN2A), receptors (e.g., the N-methyl-D-aspartate-type glutamate receptor NR2A subunit GRIN2A), synaptic proteins (e.g., PRRT2 synaptic release) and brain development pathways (e.g., mTOR) have also been proposed. Anti-epileptic drugs (AEDs) mechanism of action aims to control neuronal hyper-excitation by modifying ion channels functioning (8). Recent studies in both developed and developing countries have shown that up to 70% of epilepsy patients can be successfully treated (i.e., their seizures completely controlled) with AEDs (9, 10). But these drugs produce undesired secondary effects and in 30% of patients they are ineffective, highlighting the need to develop new alternative treatments (9, 10) aimed at alternative cellular mechanisms acting to stabilize neuronal activity.

In many developing countries, particularly in Africa and Asia, phenobarbital is the most commonly first-line prescribed AED as recommended by the World Health Organization (WHO) (7, 11–13), and this is likely because the other proven AEDs phenytoin, carbamazepine, and valproate are up to 5, 15, and 20 times more expensive, respectively (14–16). Despite the fact that access to medicines can cost as little as US$5 per year, three quarters of patients with epilepsy have no access to treatment (1). The availability and accessibility of Traditional Medicine (TM) means that it plays an important role in meeting the demands of primary health care. This was recognized by the 2008 WHO Congress on Traditional Medicine in Beijing (17), which resolved to promote the wider role of TM in worldwide health care and declared that TM should be “further developed based on research and innovation” (17).

The wild African custard-apple, *Annona senegalensis* (Magnoliales: Annonaceae) is commonly found in savannas throughout tropical Africa. Also known as *soursop, dorgot* (Wolof) and *sunkungo* (Mandinka), a decoction of its leaves and roots is used by rural African communities as a TM for the treatment of seizures, suggesting that the preparation has anticonvulsant and/or sedative properties (18). Studies have shown that extractions from root or stem have mild anticonvulsant properties on chemically induced seizures in rodents, supporting a possible role of this TM in the treatment of epilepsy (19–24). These experiments are very promising, but the screening of drugs in rodents is too costly to test the majority of ethnobiologically important candidates. Furthermore, follow-up studies investigating the pharmacological properties of novel drug candidates are intricate and even more expensive. There is therefore a need to develop a reliable, cost-effective and high throughput method for the screening of TMs with apparent anti-seizure properties that could be implemented prior to testing in animal models and clinical trials.

Adult *Drosophila* flies represent a genetically accessible and behaviorally tractable model for the study of seizures (25–27). Its strength resides in the high evolutionary conservation of most molecules controlling neural function (28). In particular, ion channels and synaptic transmission machinery proteins are largely comparable (29). *Drosophila* and humans also share several similarities in seizure phenotype thereof: (i) all individuals have a seizure threshold; (ii) seizure susceptibility can be modulated by genetic mutations; (iii) seizure activity threshold is increased by a previous electroconvulsive shock treatment; (iv) seizure activity spreads through the central nervous system (CNS) along particular pathways; (v) there is a spatial segregation of seizure activity into particular regions of the CNS; and (vi) seizure phenotypes in flies can be ameliorated by several AEDs used in humans including sodium valproate, phenytoin, gabapentin, and potassium bromide (25).

Furthermore, because *Drosophila* presents little gene redundancy, it offers a unique opportunity to study human mutated genes in an animal model (30, 31). Using CRISPR/Cas9 (32) it is easy to generate knock-in mutants where the endogenous copy of the gene is replaced by a mutated version of the human homolog, and these flies can then be used for drug testing to reveal behavioral, physiological and molecular effects (30, 31).

In this study we evaluated, for the first time, the effect of a non-commercially available drug for the treatment of seizure in *Drosophila* adult flies. We tested the hypothesis that *A. senegalensis* leaf and stem bark extracts affect the seizure patterns present in *para*^*bss*^ mutant adult flies (33, 34). We showed that *A. senegalensis* stem bark extract was significantly more effective than a leaf extract and similar to phenytoin in the prevention and control of seizure-like behavior. These results support that *Drosophila* is a robust and sensitive animal model that can be used to screen pharmacologically untested compounds which, combined with centuries of transmitted knowledge about anti-convulsant TMs, could expedite the development of the most effective TMs into novel AEDs for patients irresponsive to classical treatments.

## Materials and Methods

### Identification and Extract Preparation of *Annona senegalensis*

*Annona senegalensis* leaves and root barks were collected from “Boroboro,” 5 Km from Lira Municipality along Soroti road, Northern Uganda. Geographic coordinates are 2.190341, 32.929115 (2011'25.2”N 32055'44.8”E). Plant was identified/deposited at Department of Biology Mbarara University of Science and Technology, Uganda and given a voucher No. Moses Odur 002.

The leaves and bark removed from stem were dried and subjected to an aqueous extraction method at the Department of Pharmacology laboratory (School of Health Sciences, Kampala International University Western Campus, Ishaka-Bushenyi, Uganda). Specifically, dry leaves and stem were grounded using a blender to obtain 200 g of powder which was mixed with 1 l of distilled water in a sterile conical flask. The mixture was placed on a shaker for 72 h and then sieved to remove debris. The remaining liquid was filtered by gravity using Whatman no. 1 filter paper. The filtrate was incubated at 35°C for 1 week to evaporate the water and obtain a dry powder (35). The powder extract, approximately 10 g, was stored at 4°C until the behavioral experiments were performed.

A phytochemical analysis was performed on the aqueous extracts of the leaf and stem bark at the Kampala International University Biochemistry department lab (Supplementary Tables 1, 2).

### Drug Preparation

Given the concentration of the active compound in the aqueous *A. senegalensis* extract or the effective dose was unknown, the initial assessments were done using a high concentration (26.66 mg.ml^−1^). Animals treated with this extract solution appeared healthy and showed less seizure-like behavior than controls. We decided to expand the analysis to lower extract concentrations. *A. senegalensis* leaf and bark stem extracts and phenytoin were administered by mixing them with standard cornmeal food, which consists of 420 g of cornmeal; 450 g of dextrose; 90 g of yeast; 42 g of agar; 140 ml of 10% Nipagin in 95% EtOH; 22 ml of propionic acid and 6.4 l of water. Drug and extract solutions were prepared fresh before each experiment. Experimental doses are expressed as mg of compound per ml of food. For 13.33 and 26.66 mg.ml^−1^, the powder leaf or bark stem extract was dissolved in distilled water in a beaker at room temperature. Warm food (<60°C) was then added and mixed before aliquoting approximately 1.5 ml per testing vial. For the low concentration doses of 0.26, 1.33, and 2.67 mg.ml^−1^ a stock solution of 2.67 mg.ml^−1^ was used.

For phenytoin, one 100 mg tablet (Tophen; Agog Pharma Ltd, India) was suspended in 1 ml of distilled water, and 50, 250 μl or 500 μl of drug suspension were then mixed with 5 ml of food resulting in the low and high concentrations, respectively: phenytoin 0.909, 4.76, and 9.09 mg.ml^−1^.

### Flies Treatment and Behavioral Analysis

Flies of the bang sensitive family, specifically *bang senseless* (*bss1*) mutants of the paralytic gene *para*^*bss*1^ and easily shocked (*eas*^2*F*^) were obtained from Prof Richard Baines laboratory (University of Manchester, UK), were used (26). Oregon R (OrR) is a wildtype strain that served as a control.

Young adult flies (6–9 days) were anesthetized by cold exposure in a freezer (−20°C), separated on a cold plate into males and females and assigned to vials with 10 animals each. The flies were then randomly assigned to the different treatments vials, (standard cornmeal food with or without drug/extract). Each treatment was tested in at least 3 different days with flies from different cultures.

**Figure 1:**
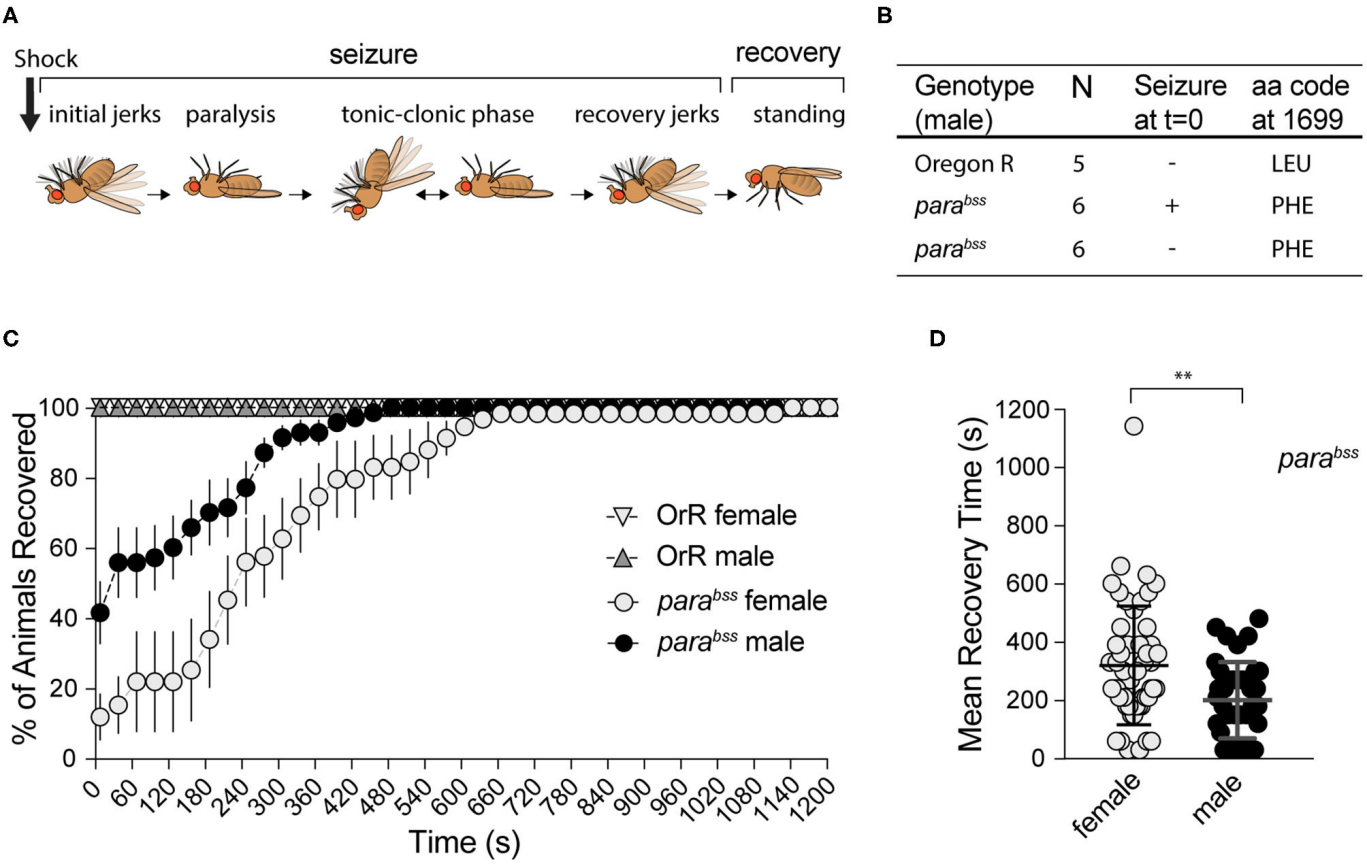
Effect of mechanical shock on seizuring behavior for female and male adult flies of *para*^*bss*^ or OrR genetic background. **(A)** Schematic of the different phases of seizure after a mechanical shock [modified from Parker et al. (38)]. **(B)** Result of *para*^*bss*^ gene sequencing in seizuring and non-seizuring males to test for the presence of the PHE mutation in position 1699 of the protein. All flies have the mutation. **(C)** Mechanical stimulation did not induce seizuring in OrR flies. *para*^*bss*^ flies showed high proportion of seizuring at *t* = 0, the percentage of flies recovered increased as a function of time. The graph shows the mean % of flies recovered ± SEM [*para*^*bss*^ females *n* = 6 (60 flies); *para*^*bss*^ males *n* = 7(70 flies); OrR females *n* = 3 (30 flies); OrR males *n* = 3(30 flies)]. **(D)** Mean recovery time comparing males and females *para*^*bss*^ (_*Mann*−*Whitney*_*U* = 667, *n*_females_ = 51, *n*_males_ = 41, *P* = 0.0034 two-tailed).

Flies were allowed to feed for 24 h prior to behavioral manipulations. On the day of behavioral assessment, flies were gently transferred into empty vials and immediately mechanically stimulated by placement on a bench-top vortex at maximum speed for 10 s (36, 37). Number of flies per vial on their backs, paralyzed, shaking or standing were recorded at 30 s intervals for 20 min. Paralyzed and shaking behaviors were recorded as seizures, whereas fully recovered flies were defined as showing standing behavior. Number of replicates and number of flies tested is detailed in Figure 1 legend and in Table 1. A few flies died before testing (sticking to the food during transfer or the 24 h drug treatment) and we had counted 1 or 2 extra flies in a few tubes. The average number of female flies tested for each treatment compared with the control was not different apart for female leaf 0.27 mg.ml^−1^ and 1.33 mg.ml^−1^ [ANOVA *F*_(13, 101)_ = 4.20, *p* < 0.0001, η^2^ = 0.35; Dunnett's multiple comparisons test *p* = 0.008 and *p*= 0.01, respectively]. No differences were registered in males [*F*_(13,95)_ = 1.65, *p* = 0.09, η^2^ = 0.18]. This supports that survival of flies was not affected by drug or extract treatment condition. We aimed to have 8 tube replicas for each extract or drug concentration but labeling errors between male and female flies produced variability on the final sample size.

**Table 1:** Prophylactic effect of the Drug and Extract Treatments in *para*^*bss*^ flies.

**Treatment** ***para^**bss**^* flies**	**Concentration** **(mg.ml-1)**	**% of recovery at** ***t*** **= 0** **Females**			**% of recovery at** ***t*** **= 0** **Males**		
		**(Mean ± SEM)**	***p***	***n* (# flies)**	**(Mean ± SEM)**	***p***	***n* (# flies)**
Control	0	15.85 ± 4.71		12 (134)	53.95 ± 4.07		9 (89)
Phenytoin	0.91	66.13 ± 9.55	<0.0001	8 (71)	89.86 ± 3.28	<0.0001	8 (79)
	4.76	54.67 ± 10.68	0.01	5 (56)	91.67 ± 3.33	<0.0001	8 (76)
	9.09	56.13 ± 4.82	0.003	6 (59)	83.15 ± 5.12	0.001	6 (51)
*A. senegalensis*	0.27	34.88 ± 5.06	0.39	8 (68)	75.87 ± 3.88	0.01	8 (75)
Leaf extract	1.33	45.36 ± 4.52	0.015	9 (78)	78.7 ± 6.64	0.01	6 (57)
	2.66	45.39 ± 4.17	0.25	8 (79)	66.25 ± 5.98	0.31	8 (80)
	13.3	58.84 ± 7.70	0.04	8 (89)	71.94 ± 4.80	0.07	8 (77)
	26.6	51.34 ± 7.96	0.006	9 (89)	76.28 ± 6.65	0.01	8 (75)
*A. senegalensis*	0.27	64.20 ± 10.56	<0.0001	8 (72)	86.83 ± 5.53	<0.0001	8 (78)
Stem Bark extract	1.33	71.77 ± 5.97	<0.0001	7 (62)	89.89 ± 3.61	<0.0001	7 (65)
	2.66	45.39 ± 4.17	0.02	9 (88)	86.25 ± 3.75	<0.0001	8 (80)
	13.3	44.42 ± 7.55	<0.0001	9 (92)	74.21 ± 6.24	0.03	8 (82)
	26.6	49.09 ± 7.25	0.001	8 (78)	77.47 ± 4.97	0.005	9 (92)

*The percentage of recovery of para^bss^ flies was evaluated at t = 0, just at the end of the mechanical stimulation. ANOVAs showed effect of treatment in female [F_(13, 114)_ = 4.48, p < 0.0001] and male flies [F_(13, 108)_ = 4.74, p < 0.0001]. Dunnett's post hoc test comparing each treatment against the control for each sex is shown in column p. The n (# flies) column shows the number of replicates (n) and flies tested (in brackets) per condition*.

Mean recovery time was calculated as the mean of the time it took for each individual that showed a bang sensitive phenotype to complete recovery. Flies that never seized were not included and animals that had not completely recovered at the end of the experiments were included as 1,200 s.

### Sequencing of *paralytic* Gene

Male flies were given a vortex shock to induce seizures as describe above. Seizing males were separated from non-seizing males. The DNA of individual *para*^*bss*^ and OrR flies was extracted using microLysis plus (Clent-Life Science), part of gene amplified using F1: TGTCAAGTGTTTATGTCTCGAGC and R1: CAGATGTTGAACAGGGCCG and sequenced using F2: TCCAGGAGCTTTAGTCGCC and R1 primers that allow to amplify the region that encodes the amino acid in 1699 which is mutated in *para*^*bss*^ mutants (38).

### Gut Content Quantification

Groups of 10 flies were fed with the extract solutions plus 0.5% (w/v) bromophenol blue salt (B5525, Sigma) for 24 h, as in the behavioral experiments. Sub-groups of 3 flies where homogenized in 30 μl of PBS (1x) with a plastic pestle in a 1.5 ml Eppendorf tube, centrifuged twice at 13,000 rpm for 2 min (39). Dye content of the second supernatant was quantified at 594 nm using a NanodropOne spectrophotometer (Thermo Scientific). The background absorbance was obtained processing groups of flies reared in the same manner but on dye-free food. The gut food content was calculated based on a standard curve done with serial dilutions of 0.5% (w/v) bromophenol blue in PBS.

### Seizuring Data Normalization

Seizure data for each sex was normalized independently. Mean recovery of control flies (without extract/drug) at *t* = 0 (*Rc*_t0_) was considered as 0% recovery, and used to normalize recovery for each experimental condition at *t* = 0 (Rti_t0_). Therefore, recovery normalized was calculated as R_norm_ = (Rti_t0_–*R*c_t0_)/(100–*Rc*_t0_) × 100. Normalized values were used to calculate mean and SEM.

### Statistical Analysis

All behavioral data was expressed as the mean % of flies per experimental vial ± SEM. Mixed ANOVAs, with group (sex or treatment) as the between-subject factor and time as the within-subject factor, were used to analyse recovering behavior. If the ANOVA showed a significant effect of time, drug treatment or the interaction between time and drug treatment, we performed a Dunnett's *post hoc* test to compare between sexes and/or drug concentrations. An ANOVA and Dunnett's *post hoc* test comparing treatments was applied to evaluate differences at *t* = 0.

Because the distribution of MRT is not normal (as tested with D'Agostino & Pearson's normality test), a Kruskal-Wallis test followed by Dunn's multiple comparisons test to compare the MRT for each drug treatment to the control was done. A two-way ANOVA with *post hoc* Dunn's multiple comparisons test was performed to analyse the food intake experiment. The α level was set at 0.05 for all analyses.

### Biosecurity Statement

The animal facility, flyroom, is a secure facility with access only granted to researchers who have had an induction to the animal facility covering health and safety and biosecurity arrangements. Access is through a swipe card system with approval only given on completion of the induction. To prevent animals escaping, the flyroom windows are sealed and the doors remain closed except when people are coming in and out and when moving equipment. In all cases there are at least three doors between the flyroom and the outside to further minimize the risk of animals escaping from the facility.

## Results

### Behavioral Characterization of *para^*bss*^* Mutants

In order to evaluate the effect of *A. senegalensis* extracts on seizures, we first characterized the seizure-like phenotype (from now on referred to as seizuring) of our *para*^*bss*^ mutant strain. *para*^*bss*^ is a bang-sensitive mutant, extremely sensitive to seizures that are characterized by 6 phases (Figure 1A). In response to a mechanical shock, many flies become immediately paralyzed while others have a short period of shaking accompanied by leg twitching, abdominal muscle contractions, rapid wing flapping and proboscis extensions before paralysis. In a fraction of animals' paralysis is interrupted by muscle jerks, in what has been described as a tonic-clonic phase. Most flies recover within 10 min following the mechanical shock, some of them after a short period of recovery shaking. After 20 min all animals are capable of maintaining their standing posture and can walk or fly (38) (Figure 1A).

The seizure phenotype observed in *para*^*bss*^ is due to a gain of function mutation, L1699F, in the sole fly voltage gated sodium channel gene *paralytic* (38) (Figure 1B). This mutation alters the voltage dependency of channel inactivation, making neurons more excitable and increasing the risk of aberrant electrical activity and seizures (38). Mutations in the human ortholog, *SCN1A*, are associated with a wide spectrum of epilepsies with over 600 mutations registered in this sole locus (8).

To induce seizures, we performed a mechanical stimulation (10 s vortex) to *para*^*bss*^ and wildtype OrR adult flies. We evaluated behavioral patterns of female and male flies separately because *para*^*bss*^ mutation is located in the X chromosome.

Immediately after induction (*t* = 0), none of the 60 (10 per vial) wildtype OrR flies evaluated showed a seizuring phenotype, while 88 ± 5% of *para*^*bss*^ females and 59 ± 8% of males seizuring (Two-way ANOVA, *p* = 0.023; Figure 1C). As expected, after a few seconds paralyzed, flies started to recover. Some of them recovered their standing posture directly while others went through a tonic-clonic phase, showing strong wing flapping and leg shaking, before standing. Mean recovery time (MRT) for flies showing a seizure-like phenotype at the beginning of the experiment was longer for females (319 ± 28 s) than for males (201 ± 20 s; Mann-Whitney test: *U* = 677, *p* = 0.0034; Figure 1D).

A mixed ANOVA on percentage of animals recovered (Figure 1C) showed an effect of time [time: *F*_(40,440)_ = 34.03, *p* < 0.001, η^2^ = 0.76] and sex [*F*_(1,11)_ = 11.16, *p* = 0.007, η^2^ = 0.50], and an interaction [time X sex: *F*_(40,440)_ = 3.17, *p* < 0.001, η^2^ = 0.22]. These results showed an unexpected sexual dimorphism in seizuring phenotype, with males being more resistant to the initiation of seizures and recovering faster than females if and when they showed the seizure phenotype. We confirmed that our stock still carries the mutation described in the literature, sequencing the paralytic gene in OrR wildtype flies and *para*^*bss*^ mutants. All 12 mutants, 6 of which did not seize at *t* = 0, had LEU mutated to PHE in position 1699. Therefore, despite these differences, the high penetrance of seizure-like behavior and the reproducibility of the phenotype confirm that *para*^*bss*^ flies are well suited to evaluate anti-seizure properties of candidate compounds as well as the dynamics of recovery from seizuring and indicate that male and female behavior need to be assessed separately.

### Effect of *Annona senegalensis* Extracts: Female Flies

**Figure 2:**
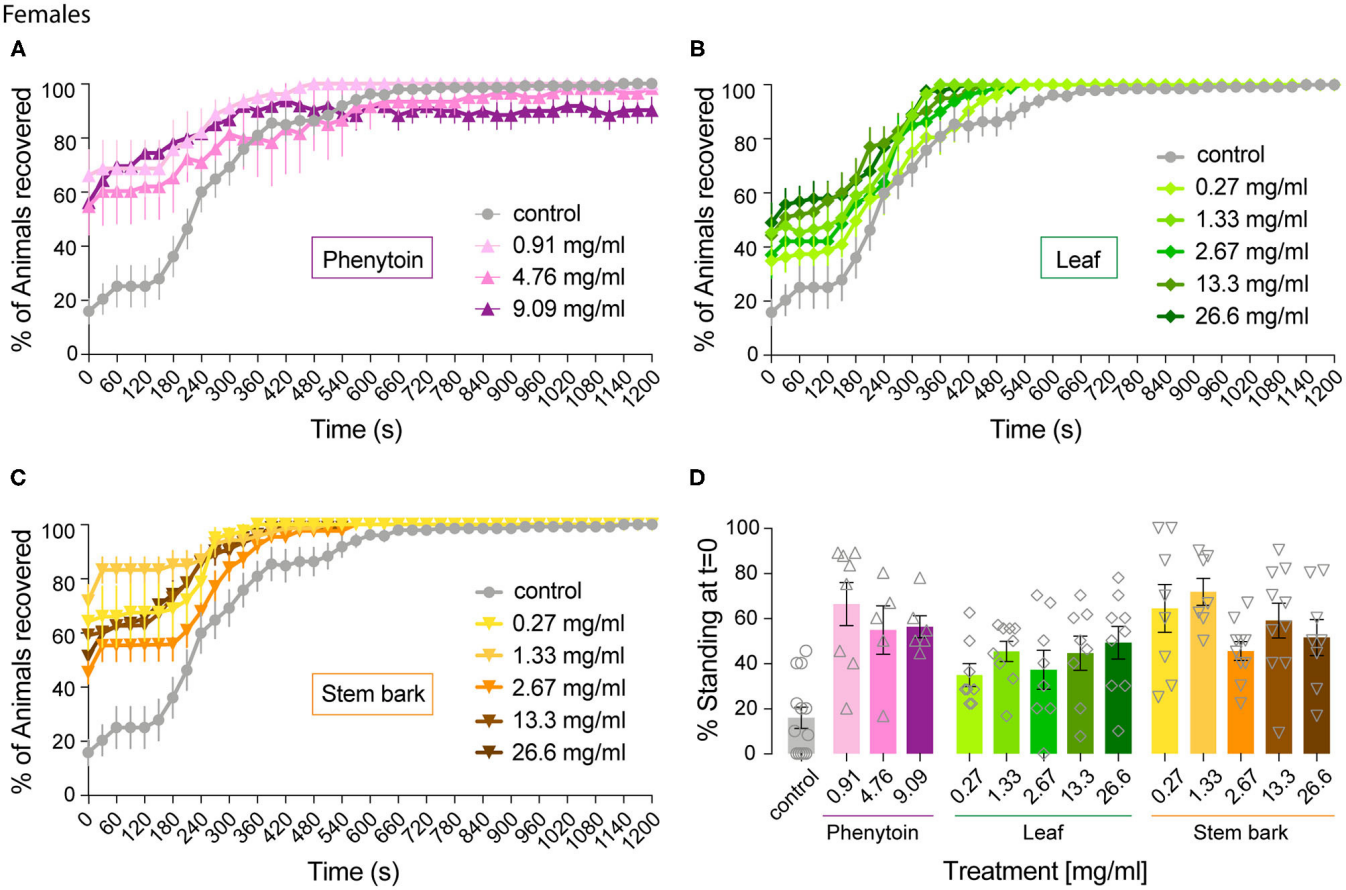
Effect of phenytoin and *A. senegalensis* leaf and stem extracts on recovery from seizures of *para*^*bss*^ female flies. *para*^*bss*^ flies treated with control food showed a very low percentage of flies recovered from seizures after mechanical perturbation, which improved over time. **(A)** Treatment with phenytoin increased the percentage of recovered flies at three different doses. **(B)** Treatment with *A. senegalensis* leaf extracts increased the percentage of flies recovered. **(C)** *A. senegalensis* stem extract doses showed a greater effect than the leaf extract. **(D)** Dose response analysis at *t* = 0 for all treatments. Graphs show the mean % of flies seizuring ± SEM (for number of replicates, flies and significance values see Table 1).

To evaluate the effect of *A. senegalensis* as an anticonvulsant, we compared the seizure-like behavior of *para*^*bss*^ flies kept in control food to animals treated with food mixed with aqueous extract from leaf or stem bark of *A. senegalensis*. As a positive control, a group of flies was treated with phenytoin, a commonly prescribed AED which has already been tested in flies (36) (Figure 2). Flies received an acute drug treatment consisting of feeding on control or treated food for 24 h before behavioral testing. Seizure-like behavior measured as the % of recovered animals across time was analyzed by mixed ANOVA comparing the effect of drug and time for female flies. An effect of time [time: *F*_(40,4040)_ = 238.83, *p* < 0.0001, η^2^ = 0.70], drug [drug: *F*_(13,101)_ = 4.53, *p* < 0.0001, η^2^ = 0.37] and an interaction [time X treatment: *F*_(520,4040)_ = 3.29, *p* < 0.0001, η^2^ = 0.30] was found in females confirming that the treatments modified the time of recovery from seizures. We therefore analyzed the individual effect of each drug treatment on the seizure-like phenotype.

**Figure 3:**
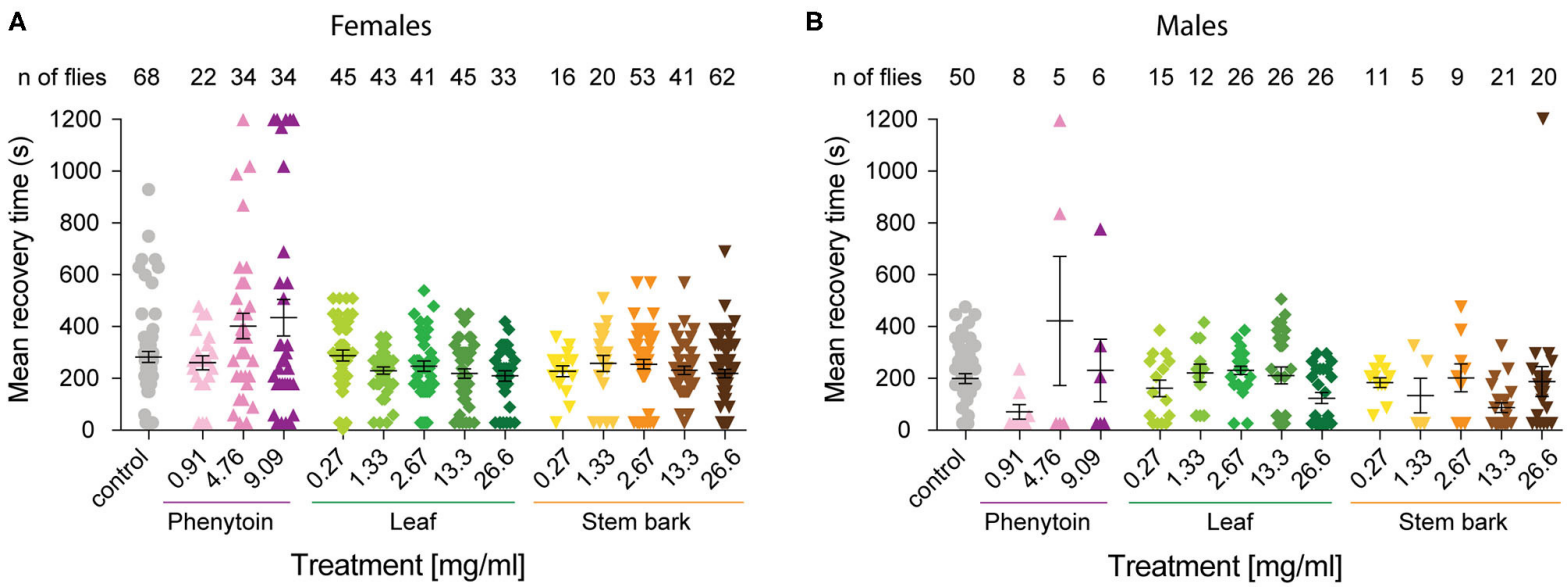
Mean recovery time for *para*^*bss*^ flies treated with phenytoin and, leaf and stem bark *A. senegalensis* extracts. The time it took for each individual that showed a bang sensitive phenotype to complete recovery was calculated. Flies not seizuring were not included and if a fly was still seizuring at the end of the experiment its time was recorded as 1,200 s. Mean ± SEM is shown. **(A)** The MRT was significantly affected by the treatment in females (Kruskal-Wallis test *H*_(14)_ = 30.21, *p* = 0.004). There were no differences between the control and other treatments as tested with a Dunn's *post hoc* test. **(B)** The MRT was significantly affected by the treatment in males (Kruskal-Wallis test *H*_(14)_ = 31.04, *p* = 0.003). Dunn's *post hoc* test showed that the control was only different with the stem bark 13.3 mg.ml^−1^ treatment (*p* = 0.02). Number of males is lower because MRT measures the time to recovery of paralyzed flies, and fewer males than females where paralyzed at *t* = 0.

As expected, *para*^*bss*^ flies kept in control food were significantly affected by the mechanical stimulation, with 15.85 ± 9.55% of flies standing at *t* = 0 and a MRT of 284 ± 21 s (Figure 3A).

As a positive control we evaluated phenytoin, a drug known to modulate the gating of voltage gated sodium channels (40). Phenytoin has already been shown to diminish seizures in *para*^*bss*^ flies, but those tests used different concentrations to the range used in our experiments and males and females were analyzed together. In the present study, phenytoin significantly improved the seizure-like phenotype of *para*^*bss*^ females over time. A mixed ANOVA on % of animals recovered in control vs. the three phenytoin concentrations showed significant interaction effects of treatment across time [time X treatment: *F*_(120,1120)_ = 5.07, *p* < 0.0001, η^2^ = 0.35] and time alone [time: *F*_(40,120)_ = 39.94, *p* < 0.0001, η^2^ = 0.59], and an effect of treatment [F_(3,28)_ = 3.69, *p* = 0.02, η^2^ = 0.28; Figure 2A]. A follow-on *post hoc* analysis showed a significant effect of phenytoin at 0.91 mg.ml^−1^ (Dunnett test: vs. control *p* = 0.007) but no effect at the higher doses, 4.76 and 9.09 mg.ml^−1^ (*p* > 0.3). At 4.76 and 9.09 mg.ml^−1^ we observed that the curves of % of recovery were below the curve of control after 480 s indicating that *para*^*bss*^ flies were recovering faster than the flies treated with the higher concentrations (Figure 2A). Indeed, phenytoin failed to shorten the MRT at 4.76 and 9.09 mg.ml^−1^ and the tendency was to increase it (Figure 3A), suggesting that the higher concentrations might have a small toxic effect on the flies. However, phenytoin had a clear prophylactic effect at *t* = 0. The three concentrations increased the percentage of recovered flies being the lower concentration the most effective (Dunnett test: control vs. 0.91 mg.ml^−1^ *p* < 0.0001; vs. 4.76 mg.ml^−1^ *p* = 0.009 and vs. 9.09 mg.ml^−1^ *p* = 0.003; Figure 2D and Table 1). At these concentrations, phenytoin protected against the initiation of seizure (Figures 2A,D, 3).

We then analyzed the effect of *A. senegalensis* leaf extract (Figures 2B,D). The five curves of animals treated with the leaf extract showed an improvement in the percentage of flies recovered from seizure over time. This observation was confirmed by a mixed ANOVA on the percentage of flies recovered, which showed significant effects of time [time: *F*_(40,1960)_ = 189.37, *p* < 0.0001, η^2^ = 0.79], treatment [drug: *F*_(5,49)_ = 6.56, *p* < 0.0001, η^2^ = 0.40] and their interaction [time X drug: *F*_(200,1960)_ = 3.07, *p* < 0.0001, η^2^ = 0.24]. A follow-on Dunnett's *post hoc* analysis comparing each drug with the control showed no effect at 0.27 mg.ml^−1^ (*p* = 0.31) and a greater time-dependent recovery in animals treated with the leaf extract above 1.33 mg.ml^−1^ (control vs.: 1.33 mg.ml^−1^ *p* < 0.0001; 2.67 mg.ml^−1^ *p* = 0.05; 13.3 mg.ml^−1^ *p* = 0.002 and 26.6 mg.ml^−1^ *p* = 0.001). The quantification at *t* = 0 showed an improvement in the percentage of flies insensitive to seizure depending on the concentration with a significant prophylactic effect at 0.27, 1.33, 13.33, and 26.6 mg.ml^−1^ (Figure 2D and Table 1). To test if the leaf extract alleviates the seizure by reducing their duration, we quantified the MRT. There was no shortening of the MRT comparing the *para*^*bss*^ control to the five concentrations of leaf extract (Kruskal- Wallis with Dunn's *post hoc* test; Figure 3A). All together, these results indicate an anti-seizure effect of *A. senegalensis* leaf extract on *para*^*bss*^ female flies that is mainly attributed to a prophylactic effect of the drug.

Finally, we evaluated the anti-convulsant properties of *A. senegalensis* stem bark extract (Figures 2C,D). A mixed ANOVA on percentage of flies recovered showed significant effects of time [time: *F*_(40,1920)_ = 105.02, *p* < 0.0001, η^2^ = 0.69], treatment [*F*_(5,48)_ = 9.67, *p* < 0.0001, η^2^ = 0.50] and of their interaction [time X treatment: *F*_(200,1920)_ = 5.05, *p* < 0.0001, η^2^ = 0.35]. A follow-on *post hoc* analysis comparing the different concentrations to the control showed that the stem bark extract treatment produced a very significant improvement of the % of recovery (Dunnett test: control vs. 0.27, 1.33, 13.33, and 26.6 mg.ml^−1^ *p* < 0.0001 and 2.6 mg.ml^−1^ *p* = 0.006). Furthermore, the five concentrations of the drug showed a significant increment of the % of flies recovered compared to control at *t* = 0 (Figure 2D and Table 1). However, this differences over time were not reflected in shorter MRTs (Figure 3A). Meaning that if a fly has started seizing it will recover on average at the same pace as a *para*^*bss*^ mutant which was not treated with the stem extract. As for the other drugs tested, the improvement in seizure-like behavior quantified in flies treated with the stem bark was mainly due to its prophylactic effect.

### Effect of *Annona senegalensis* Extracts on Male Flies

We then evaluated the seizure phenotypes of males exposed to the same treatments as females. In males under control conditions, the percentage of flies recovered after mechanical stimulation is significantly higher than in females (200 ± 21 s; Figure 1C) and the MRT is significantly shorter (204 ± 19 s; Figure 3B). Significant drug effects were therefore expected mainly at the early time points of behavioral analysis. A mixed ANOVA on the percentage of male flies recovered after mechanical stimulation showed an effect of time [time: *F*_(40,3800)_ = 96.55, *p* < 0.0001, η^2^ = 0.50], an interaction between time and treatment [time X treatment: *F*_(520,3800)_ = 108.97, *p* < 0.0001, η^2^ = 0.32], and an effect of treatment alone [treatment: *F*_(13,95)_ = 4.41, *p* < 0.0001, η^2^ = 0.38].

**Figure 4:**
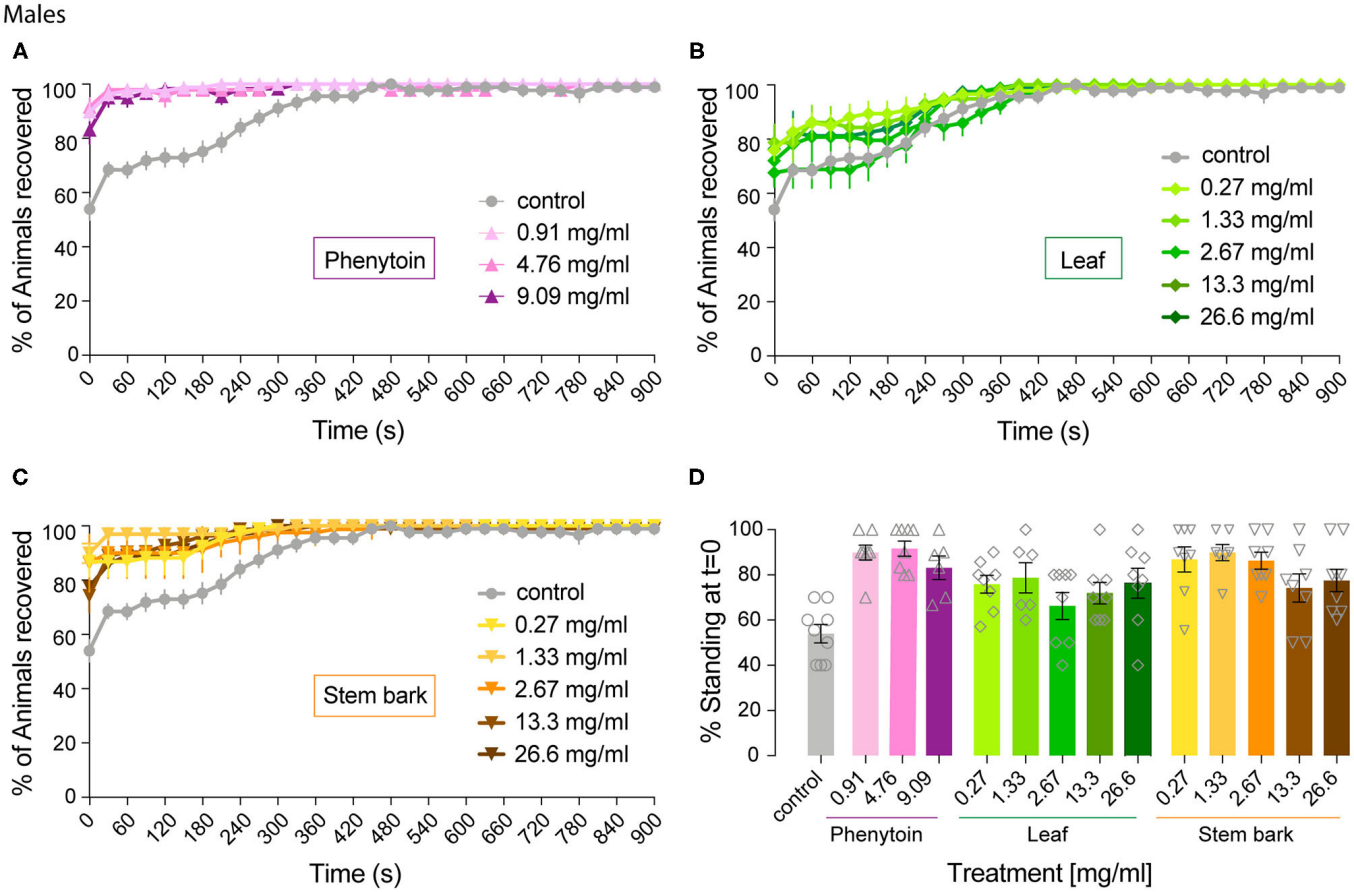
Effect of phenytoin and *A. senegalensis* extracts on recovery from seizures of *para*^*bss*^ males. Mechanical stimulation of *para*^*bss*^ control male flies induced a seizure-like phenotype that was less penetrant than in females. The effects of phenytoin **(A)**, *A. senegalensis* leaf **(B)** or stem extract **(C)** are shown. **(D)** Dose response analysis at *t* = 0 for all treatments. Graphs show the mean % of flies seizuring ± SEM (for number of replicates, flies and significance see Table 1).

We then analyzed the behavioral effect for each drug treatment independently. A mixed ANOVA on percentage of flies recovered comparing phenytoin treated animals with controls showed an effect of time [time: *F*_(40,1080)_ = 30.95, *p* < 0.0001, η^2^ = 0.53], an interaction between time and treatment [time X treatment: *F*_(120,1080)_ = 12.40, *p* < 0.0001, η^2^ = 0.58] and of treatment [*F*_(3,27)_ = 21.94, *p* < 0.0001, η^2^ = 0.71]. Further *post hoc* analysis showed that all three concentrations were significantly better than the control (Dunnett: control vs. 0.91, 4.76 and 9.09 mg.ml^−1^ of phenytoin *p* < 0.001; Figure 4A). Significant behavioral differences (*p* < 0.001) were found at *t* = 0 for the three concentrations tested (Figures 4A,D and Table 1). We did not observe differences in MRT across groups, probably due to a low number of flies seizuring at early time points. However, as observed in females, at 4.76 and 9.09 mg.ml^−1^ of phenytoin, a few flies had longer MRT then in controls, suggesting that the highest concentrations tested have a slight toxic effect on male flies as well (Figure 3B).

We then evaluated the effect of *A. senegalensis* leaf extract treatment. A mixed ANOVA on the percentage of flies recovered showed a significant effect of time [time: *F*_(40,1640)_ = 68.50, *p* < 0.0001, η^2^ = 0.63], and a significant interaction between time and drug treatment [time X treatment: *F*_(200,1640)_ = 1.46, *p* < 0.0001, η^2^ = 0.15], but no effect of drug alone [treatment: *F*_(3,29)_ = 1.77, *p* = 0.14, η^2^ = 0.18; Figure 4B]. The lower effect of the leaf extract on males was further supported by a generalized more modest improvement at *t* = 0 (Figure 4D and Table 1). The MRT was not significantly improved for any concentration of the leaf extract (Figure 3B).

An effect of time, treatment and their interaction were present after treatment with stem bark extract of *A. senegalensis* in males [time: *F*_(40,1720)_ = 48.57, *p* < 0.0001, η^2^ = 0.53; treatment: *F*_(5,43)_ = 6.84, *p* < 0.0001, η^2^ = 0.44; time X treatment: *F*_(200,1720)_ = 4.10, *p* < 0.0001, η^2^ = 0.32]. A Dunnett's *post hoc* analysis confirmed the improvement in the % of recovery of the flies treated with the stem bark extract compare to the untreated control, all concentrations being highly significant (control vs. 0.27, 1.33, and 26.6 mg.ml^−1^ *p* < 0.0001 and vs. 2.67 mg.ml^−1^ *p* = 0.001 and 13.3 mg.ml^−1^ *p* = 0.002; Figure 4C). As in phenytoin treated animals, stem bark extracts very significantly decreased the percentage of animals seizuring at *t* = 0 pointing to a prophylactic effect against the initiation of seizure-like behavior (Figure 4D and Table 1). Furthermore, the treatment with stem bark extract at 13.3 mg.ml^−1^ was the only treatment to significantly decrease the MRT (*p* = 0.02; Figure 3B) supporting the effectiveness of the drug.

**Figure 5:**
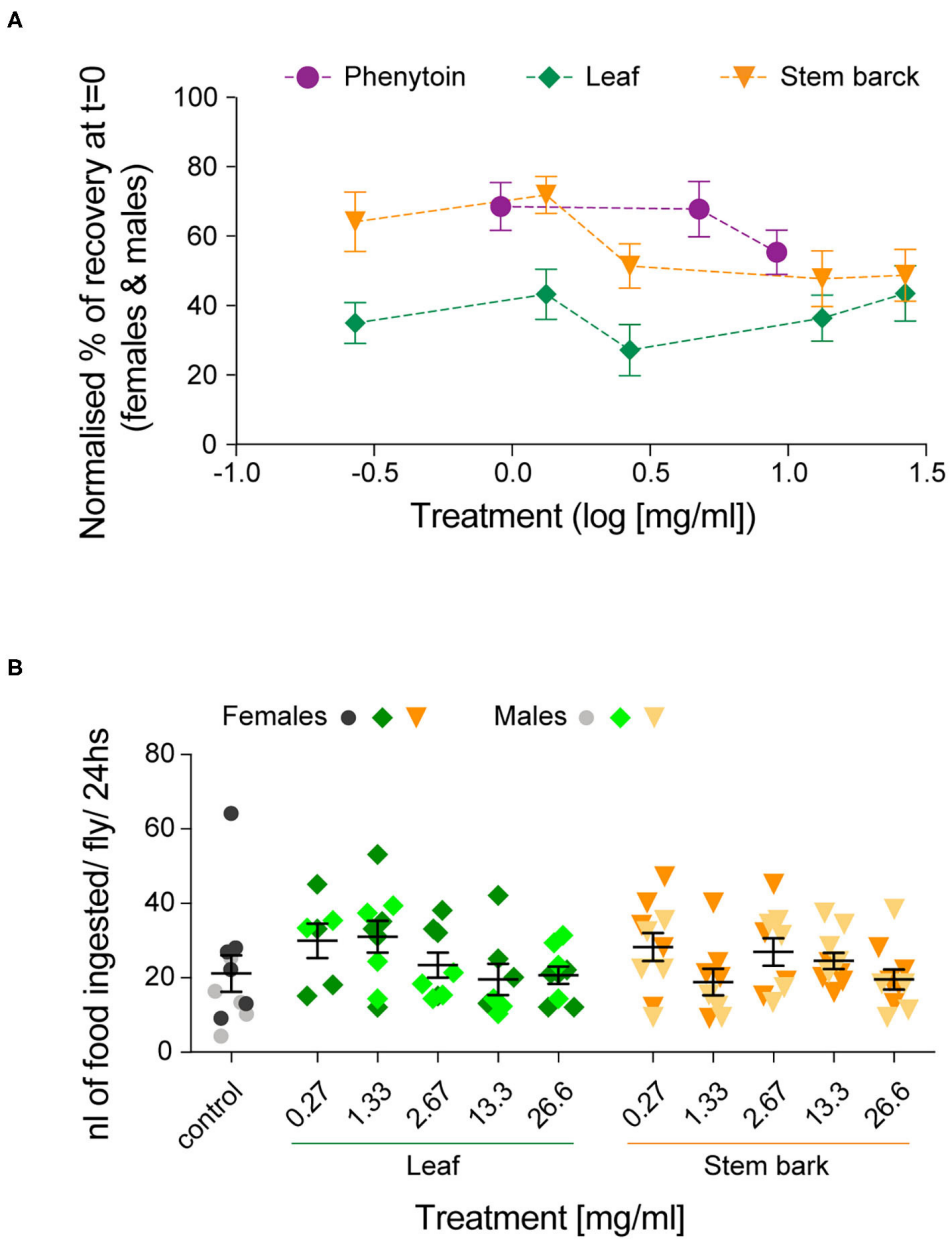
Dose-dependent prophylactic effect of phenytoin and, leaf and stem bark *A. senegalensis* extracts. **(A)** Normalized % of recovery at *t* = 0 for females and males. The concentration is in log_10_ scale. An ANOVA comparing the effect of the three drugs treatment was significant [*F*_(2,199)_ = 15.06, *p* < 0.0001, η^2^ = 0.13]. A *post hoc* Bonferroni multiple comparisons test was used to test for differences between the drugs (phenytoin vs. leaf *p* < 0.0001; vs. stem *p* = 0.35 and leaf vs. stem *p* = 0.0001). No significant differences between concentrations of each drug were found, apart when comparing with the control (Tukey's multiple comparisons test). **(B)** Amount of food ingested per fly and per 24 h. A two way ANOVA showed no effect of drug [*F*_(1,103)_ = 0.03, *p* = 0.86], no effect of concentration [*F*_(5,103)_ = 1.63, *p* = 0.16] and no interaction [*F*_(5,103)_ = 1.26, *p* = 0.28]. A *post hoc* Dunnett's multiple comparisons test comparing the control with each treatment did not yield any significant differences.

Altogether, these results show that recovery in both female and male flies was improved by treatments with *A. senegalensis* stem and leaf extract as well as with the commonly used AED phenytoin. To gain a better insight into the dose-response curve, we normalized the percentage of recovery. This allowed us to only evaluate the effect of the drug and extracts on flies seizuring at *t* = 0 (now at *t* = 0 there is 0 ± SEM % recovery for all treatments; Figure 5A). In this condition the sexual dimorphism is no longer present, and we therefore pooled the data of both sexes. The three curves are in the plateau phase and no significant effect of concentration was found, apart for when comparing with the control. At saturated doses, the prophylactic effect of phenytoin is comparable to *A. senegalensis* stem bark administration (*p* = 0.66; Figure 5A). There is a slight but non-significant decrease of phenytoin and stem bark effects, suggesting that higher concentrations of these compounds may have toxic or sedative effect. The leaf extract of *A. senegalensis* showed a lower protective effect than phenytoin (*p* < 0.0001) and stem bark (*p* = 0.0002), present at all concentrations (Figure 5A). Differences of prophylactic effects are not explained by differences in amount of food ingested since there were no significant differences in gut content between treatments (Figure 5B). The strong effect of *A. senegalensis* stem extract suggests the existence of an AED compound that could offer an alternative for the treatment of intractable epilepsy.

### Effect of *Annona senegalensis* Extracts on *easily shocked* Mutants

To test the efficacity of *A. senegalensis* when the molecular cause of the hyperexcitability is other than mutations of an ionic channel, we decided to evaluate its effect on *easily shocked* mutants (*eas*^2*F*^), which carry a mutation in an ethanolamine kinase which affects the phospholipid composition of neuronal membranes (41). This bang sensitive mutant is known to improve upon treatment with phenytoin but is partially or completely insensitive to other drugs like carbamazeprine, ethosuximide, and vigabactrin (36). When we tested the leaf and stem bark extract at 2.67 and 26.6 mg.ml^−1^ on *eas*^2*F*^ flies, we did not observe any improvement in their percentage of recovery (Table 2). Rather the mutants were more paralyzed and the only significant changes in the MRT were due to a delay in recovery (Table 2).

**Table 2:** Effect of *Annona senegalensis* treatments on the seizure-like phenotype of *eas*^2*F*^ flies.

**Treatment *eas^**2F**^* flies**	**Concentration (mg.ml-1)**	**% of recovery at** ***t*** **= 0**					
		**Females**			**Males**		
		**(Mean ± SEM)**	***p***	***n* (# flies)**	**(Mean ± SEM)**	***p***	***n* (# flies)**
Control	0	6.73 ± 3.09		13 (115)	0.63 ± 0.63		16 (137)
*A. senegalensis*	2.67	0	ns	8 (73)	1.25 ± 1.25	ns	8 (69)
Leaf extract	26.6	0	ns	6 (54)	0	ns	8 (75)
*A. senegalensis*	2.67	0	ns	8 (73)	1.39 ± 1.39	ns	8 (73)
Stem Bark extract	26.6	1.39 ± 1.39	ns	10 (92)	4.26 ± 2.11	ns	8 (74)
		**Mean recovery time**					
Control	0	50.86 ± 2.73		105	79.17 ± 3.27		133
*A. senegalensis*	2.66	59.59 ± 3.58	ns	73	83.33 ± 4.83	ns	72
Leaf extract	26.6	70.00 ± 3.72	<0.001	54	81.20 ± 4.85	ns	92
*A. senegalensis*	2.66	58.29 ± 3.39	ns	70	77.67 ± 4.68	ns	73
Stem Bark extract	26.6	66.49 ± 3.71	0.001	74	70.87 ± 4.55	ns	69

*Percentage of recovery at t =0. Kruskal-Wallis tests comparing each treatment were non-significant for females [$\chi_{\;\left(5\right)}^{2}$ = 6.45, p = 0.17] and males [$\chi_{\;\left(5\right)}^{2}$ = 7.66, p = 0.1]. The result of Dunn's post hoc test comparing each treatment against the control for each sex is included in the p column of the table. The n (# flies) column shows the number of replicates (n) and flies tested for each treatment*.

*Mean recovery time. Kruskal-Wallis test comparing each treatment showed a significant effect for females [$\chi_{\;\left(5\right)}^{2}$ = 23.98, p < 0.0001] but not for males [$\chi_{\;\left(5\right)}^{2}$ = 4.34, p = 0.36]. The result of Dunn's post hoc test comparing each treatment against the control for each sex is included in the p column of the table. The number of flies seizuring at t = 0 is shown in column n*.

This experiment shows that despite the similarity in the prophylactic effect between phenytoin and *A. senegalensis* extract on *para*^*bss*^, it is likely that their mode of action differs.

## Discussion

Approximately 30 AEDs are currently approved for treatment of patients affected by epilepsy, an aetiologically complex and variable disease. A non-negligible percentage of epileptic experience undesired secondary effects of these AEDs, and 30% of patients are insensitive to them altogether (9, 10), highlighting the necessity of discovering alternative compounds to incorporate in the design of novel therapies.

Almost all commonly prescribed AEDs were discovered by animal screenings (40) and their mechanisms of action were also studied in non-human species, emphasizing the importance of animal testing for drug discovery in epilepsy. Medicinal plant extracts have been historically used for the treatment of various diseases, including epilepsy. The knowledge gathered and transmitted for generations about the anti-seizure properties of herbal TM preparations constitute a rich source of information for the discovery of new drug candidates. However, an empirical testing of their efficacy as AED and the pharmacological characterization of the active compound is required. A few hundred drugs used in TM have been tested in epilepsy animal models but the lack of follow up clinical and pharmacological studies places constraints on the clinical recommendation of herbal TMs (42).

In the present study, we used a *Drosophila* seizure model to test the effect of *A. senegalensis* extracts as an anti-seizure treatment (25–27). We used a fly with a gain of function mutation in the unique voltage gated sodium channel of *Drosophila* genome, *para*^*bss*^, which is extremely prone to seizures. *para*^*bss*^ mutant flies are generally accepted as a model for intractable epilepsy due to the complexity of their seizures, including a tonic-clonic-like phase, and their significant resistance to AED treatments (38). *A. senegalensis* was used because of its strong reputation as a TM potentially effective for the treatment of seizures and because several studied performed in rodents have tested its anticonvulsant properties (19–24). In these studies, extracts of *A. senegalensis* were prepared with different methods (in general with polar solvents) and from different parts of the plant (leaf, root or stem). In all cases, an acute treatment with the extract, via intraperitoneal injection or via oral administration, produced a significant prophylactic effect against seizures induced by pentylenetetrazole, picrotoxin, pilocarpin or maximal electroshock (19–24). However, drug testing in mammals is costly and slow, reducing the number of compounds that can be subject to thorough analysis. Using *Drosophila* as a model system offers an opportunity to overcome such limitations and isolate active compounds in a high throughput and cost-effective manner.

*para*^*bss*^ flies showed a strong seizure-like phenotype with significant differences between males and females. Approximately 85% of females were paralyzed after the mechanical stimulation, while only 50% of the males were. Despite this level of seizuring been below the one described in previous reports (36–38), the aminoacidic residue leucine in position 1699 was mutated to Phenylalanine in our *para*^*bss*^ stock. This suggests that a repressor mutation in the X chromosome might be compensating for the L1699P mutation or, less likely, that our stock has lost an enhancer mutation that was generating the original fully penetrant phenotype.

Alternatively, this could be a consequence of the lack of controlled circadian synchronization in our experimental animals. In humans, it is known that the onset of seizures and the interictal epileptiform discharges (IED) have a tendency to occur in specific times of the day. For example, tonic-clonic seizures occur more frequently during sleep. This rhythmicity is controlled by both the circadian clock and sleep-wake state (43). In our experimental conditions, flies were inadvertently tested at different endogenous circadian time. This factor might have influenced the seizure threshold across animals, generating heterogeneity in the penetrance of the seizure phenotype. In this scenario the known behavioral differences in the rest-activity cycles between females (more active) and males might have resulted in the sexual dimorphism observed (44). It would be interesting to take advantage of the knowledge of circadian rhythms in *Drosophila* to further investigate the molecular and cellular mechanisms underlying the circadian and vigilance regulation of seizures.

Although presenting lower levels of seizuring than in the literature and sexual differences in phenotype penetrance, both sexes showed a measurable level of paralysis and recovered gradually, so they could be used to test the anti-seizure effect of *A. senegalensis*. Our experimental design was therefore suited to analyse the overall effect of drug treatments, from seizure induction to complete recovery. More precisely, we evaluated (1) the prophylactic effect against seizure induction and (2) the effect on recovery dynamics. We compared the effect of *A. senegalensis* extracts, to the commonly used AED phenytoin. Phenytoin was chosen as our positive control since its anti-convulsion properties have been documented in flies before. When administered chronically and at low doses (0.03–0.3 mg.ml^−1^) it induced a decrease in MRT in a mixed population of males and females *para*^*bss*^ (36).

In our experimental conditions (24 h drug exposure in the food, 0.91, 4.76 and 9.09 mg.ml^−1^), phenytoin produced the expected improvement on seizuring phenotype. This was characterized by a clear prophylactic effect of all three doses, with fewer male or female flies showing seizures compare to controls at the beginning of the experiment (*t* = 0). The MRT was not improved at any concentration but this might be due to a slight toxic effect of phenytoin at 4.76 and 9.09 mg.ml^−1^. At this concentration the MRT slightly increased and a few flies remained paralyzed for the entire duration of the experiment (1,200 s), a phenotype not observed in *para*^*bss*^ mutants.

The treatment with *A. senegalensis* stem bark extract effectively improved the seizure-like phenotype. At the lower doses (below 2.67 mg.ml^−1^) approximately 60% of the flies did not seizure at *t* = 0, showing a similar effect to the one quantified with phenytoin. We could not quantify a decrease in the MRT indicating that once the seizure has started the drug barely ameliorates the symptoms. In comparison, treatments with *A. senegalensis* leaf extracts were less effective than stem bark extracts, suggesting that the active compound of the plant is most concentrated in the stem bark.

These findings are significant because they lay the bases for future studies aiming to discover the active chemical compound present in *A. senegalensis* stem bark extract. In that direction, it would be possible to use this experimental approach to test the anti-seizure effect of samples coming from an extensive sub-fractioning of stem bark extracts. This would allow further purification and identification of the active compound.

One such simple sub-fractioning experiment was performed from *A. senegalensis* roots. It identified a putative candidate belonging to the diterpenoid family, Kar-16a-19oic acid, that showed promising sedative and anti-seizure properties in rodents (21). The candidate compound was enriched in an ethyl-acetate fraction and showed an acute toxicity in mice of LD_50_ = 2154 mg/kg compared to LD_50_ = 150 mg/kg for phenytoin (pubchem). Toxicity of an aqueous extract, like the one used in our experiments, in mice is lower, LD_50_ > 5,000 mg/kg (45), which would make it ideal for its clinical recommendation as a TM (9). This and other candidates could be further tested using the present animal model.

The difference in prophylactic effect observed on *para*^*bss*^ and *eas*^2*F*^ mutant flies gives an indication regarding the possible mechanism of action of *A. senegalensis* extract. It has already been shown that the percentage of *eas*^2*F*^ mutants seizuring improves when treated with phenytoin (36) but that the mutants are resistant to treatment with several AEDs (36): carbamazepine a sodium channel blocker; ethosuximide that blocks T-type calcium channels, and may include effects on other classes of ion channel; vigabatrin that increases GABA in the brain blocking the activity of the gamma-aminobutyric acid aminotransferase and gabapentin which has recently been shown to selectively inhibit calcium channels containing the a2d-1 subunit (46). Because *A. senegalensis* improves the phenotype of *para*^*bss*^ but not *eas*^2*F*^, it is likely that the active compound acts via a mechanism different than phenytoin, not acting on voltage gated sodium channels (40). On the other hand, an anticonvulsant effect of *A. senegalensis* leaf extract was shown on pentylenetetrazol (PTZ) induces convulsions in mice (19). PTZ antagonizes GABA_A_ receptor Cl– channel complex (19) attenuating GABA-dependent inhibition and inducing seizures. It is possible that *A. senegalensis* effect is partially due to enhancement of GABAergic pathways. If this were the case, a high concentration of the plant extract could produce a sedative effect and induce more paralysis as observed when testing *eas*^2*F*^. Ultimately, experiments to test the mechanism of action of *A. senegalensis* extract should be performed.

The simplicity, reproducibility, potential high throughput and low cost of the testing method reported here constitutes an ideal setup to isolate the active anti-seizure compound present in *A. senegalensis* and potentially other TMs. Molecules controlling the development and function of the nervous system (28, 29) in *Drosophila* and humans present high structural and functional conservation. *Drosophila* could provide an early neurophysiological characterization of the mechanisms of action of new candidate compounds with potential translational anti-seizure effects (25–27, 47, 48). Specific neurons whose neurotransmitter identity and connectivity are known could be patch-clamped in *para*^*bss*^ and the effect of the drug tested (49). The contribution of inherited or *de novo* mutations in the etiology of epilepsy, including focal epilepsy (6, 50), is growing. Sodium channels, that play a crucial function for cellular excitability, have been associated with a wide range of epilepsies (5). Amongst them is *SNC1A*, the human ortholog of *para*^*bss*^, with over 600 different mutations found in patients (8). Links to other ionic channels, receptors synaptic proteins and brain development pathways have also been proposed. The genetic malleability of *Drosophila*, where specific gene mutations can easily be generated, strengthens its validity for screening and early characterization of neural mechanism underlying hyperexcitability.

Screening TMs in several distinct *Drosophila* seizuring mutants will most likely help isolating new compounds for intractable epilepsy. This is exemplified by the lack of prophylactic effect induced by *A. senegalensis* extract on *eas*^2*F*^ mutants, which are insensitive to many EADs but might benefit from new drugs (36).

However, fruit flies and humans also show fundamental differences that need to be taken into consideration when interpreting screening results. From a circuit point to view, the fly brain is much simpler with no cortical and subcortical regions where most focal source of seizure are located in humans. From the aetiological perspective, the causes of epilepsy in humans are varied ranging from inherited (genetic) to acquire through brain injury (e.g., prenatal or perinatal causes, severe head or brain injury, stroke, infections) (11). Treatment with AEDs aims to re-balance neuronal activity but fly models of epilepsy might fail to capture the full spectrum of neural and brain dysfunctions. Limited behavioral phenotypes in flies suggest that only seizure resembling the “grand mal” with strong spams and muscular manifestations could be studied. More complex or subtle cognitive and emotional aspects of epilepsies are not accessible in flies. Finally, absence of a proper brain blood barrier, an open hemolymph circulation and the distinctive renal system of *Drosophila* compromises translation of pharmacokinetics and toxicity data to humans.

Ultimately, pre-clinical research in different animal models complemented with clinical trials in humans will be essential in establishing effectivity and safety profiles for TM compounds for the treatment of epilepsy.

In conclusion, our work is important because it confirms how powerful *Drosophila* is as a model system for screening of putative new AEDs. Regardless of the fact that there was little information about how to prepare the plant extract, we did not know the identity of the active compound, its effective dose, pharmacokinetic, or toxicity in flies, we were able to find a strong anti-seizure effect of *A. senegalensis* stem bark extracts on *para*^*bss*^ flies. This method could be used for high throughput screening of many TM with suspected anti-convulsion properties, hence offering a robust platform for the identification and isolation of novel AEDs that could be the basis of new treatments for intractable epilepsy.

## Data Availability Statement

The original contributions presented in the study are included in the article/Supplementary Materials, further inquiries can be directed to the corresponding author/s.

## Ethics Statement

Ethical review and approval was not required for the animal study because the use of Drosophila is not included the UK Home Office regulation.

## Author Contributions

SD and JB conceived and designed the experiments. SD, JB, JRC, and NH performed the experiments. EM and JB analyzed the data and EM did the statistics. PE identified the plants and prepared the extracts. EM and JB wrote the manuscript. All authors contributed to the article and approved the submitted version.

## Conflict of Interest

The authors declare that the research was conducted in the absence of any commercial or financial relationships that could be construed as a potential conflict of interest.
