# Supplementary material for: Drosophila parabss Flies as a Screening Model for Traditional Medicine: Anticonvulsant Effects of Annona senegalensis
Source: Front Neurol. 2021 Jan 13;11:606919. doi: 10.3389/fneur.2020.606919 (PMC7838503; doi:10.3389/fneur.2020.606919)
Supplement: Supplementary file 1 [file Table_1.DOCX]

Supplementary Material

# Supplementary Material

**S1 Table: Phytochemical Analysis of Aqueous extract of Leaf of *Annona Senegalensis***

| **S/N** | **Bioactive component** | **Methods** | **Observation** | **Leaves** |
| --- | --- | --- | --- | --- |
| 1. | Saponins | 5ml of distilled water was added to 1 ml of extract | Frothing was observed on heating and on cooling as well | + |
| 2. | Tannins | 1ml of extract was mixed with a few drops of ferric chloride | There was formation of green colour which indicates the presence of condensed tannins | + |
| 3. | Quinones | 1ml of extract was mixed with 1ml of conc. H_2_SO_4_ | A red colour formed | ++ |
| 4. | Reducing sugars | 5ml of Benedict reagent was added to 1ml of filtered extract and the mixture heated to boiling | Colour changed from blue to green | + |
| 5. | Flavonoids | Diluted NaOH was added to 1ml extract followed by diluted HCL | The yellow solution turned colourless on addition of HCl | ++ |
| 6. | Cardiac glycosides | To 1ml of extract, a few drops of glacial acetic acid were added followed by 1 drop of ferric chloride. 1 ml of conc. H_2_SO_4_ was gently added | A deep brown ring formed at the interface. This is a deoxy sugar characteristic of cardenolides | ++ |
| 7. | Alkaloids | 1ml of extract was acidified with HCL. To the mixture, a few drops of Wegner’s reagent were added | No yellow or brown precipitate was formed | - |
| 8. | Amino acids | To 1ml of extract, 3 ml of Ninhydrin reagent was added and the mixture boiled for a few minutes | The colour changed from brown to deep blue | +++ |
| 9. | Steroids and Triterpenoids | To 1ml of extract a few drops of acetic anhydride was added boiled and cooled. Small amount of conc. H_2_SO_4_ was added down side of the tube | A brown ring was formed at the junction of the two layers. A red colour also formed indicating the presence of triterpenoids | ++ |
| 10. | Terpenoids | To 1ml of extract, 2 ml of chloroform and 3 ml of conc. H_2_SO_4_ was carefully added to form a layer | A reddish brown colouration formed at the interface | + |
| 11. | Diterpenes | To 1 ml of extract, 3 drops of copper acetate solution were added | Colour changed to emerald green (faintly) | + |
| 12. | Resins | 1 ml of extract was dissolved in alcohol solution and the mixture was added to 10 ml of distilled water | No precipitation was formed (A solution formed) | - |
| 13. | Phenols | To 1 ml of extract, 2 ml of distilled water were added and followed by a few drops of 10% ferric chloride | No blue or green colour was formed | - |
| 14. | Phlobatanins | To 1 ml of extract, 2% HCL was added and the mixture was heated to boiling | No red precipitate was formed | - |
| 15. | Anthraquinones | To 1 ml of extract, 1 ml conc HCL was added followed by a few drops of 10% FeCl_3_ and diethylether. 1 ml of conc. Ammonium solution was also added | No observable change | - |

**Key: + = Present;**

**- = Absent**

**S2 Table. Phytochemical Analysis of Aqueous extract of Stem bark of *Annona Senegalensis***

| **S/N** | **Bioactive component** | **Methods** | **Observation** | **Stem Barck** |
| --- | --- | --- | --- | --- |
| 1. | Saponins | 5ml of distilled water was added to 1 ml of extract | Frothing was observed on boiling and on cooling | ++ |
| 2. | Tannins | 1ml of extract was mixed with a few drops of ferric chloride | There was formation of green colour which indicates the presence of condensed tannins | +++ |
| 3. | Quinones | 1ml of extract was mixed with 1ml of conc. H_2_SO_4_ | A red colour formed | ++ |
| 4. | Reducing sugars | 5ml of Benedict reagent was added to 1ml of filtered extract and the mixture heated to boiling | The blue colour of Benedicts solution persisted even after boiling | - |
| 5. | Flavonoids | Diluted NaOH was added to 1ml extract followed by diluted HCL | The yellow solution turned colourless on addition of HCl | ++ |
| 6. | Cardiac glycosides | To 1ml of extract, a few drops of glacial acetic acid were added followed by 1 drop of ferric chloride. 1 ml of conc. H_2_SO_4_ was gently added | A brown ring appeared at the interface which is a typical deoxy sugar characteristic of cardenolides | +++ |
| 7. | Alkaloids | 1ml of extract was acidified with HCL. To the mixture, a few drops of Wegner’s reagent were added | No yellow or brown precipitate was formed | - |
| 8. | Amino acids | To 1ml of extract, 3 ml of Ninhydrin reagent was added and the mixture boiled for a few minutes | The colour changed from brown to deep blue | +++ |
| 9. | Steroids and Triterpenoids | To 1ml of extract a few drops of acetic anhydride was added boiled and cooled. Small amount of conc. H_2_SO_4_ was added down side of the tube | A brown ring was formed at the junction of the two layers. A red colour also formed indicating the presence of triterpenoids | ++ |
| 10. | Terpenoids | To 1ml of extract, 2 ml of chloroform and 3 ml of conc. H_2_SO_4_ was carefully added to form a layer | A reddish brown colouration formed at the interface | + |
| 11. | Diterpenes | To 1 ml of extract, 3 drops of copper acetate solution were added | Colour changed to emerald green (faintly) | ++ |
| 12. | Resins | 1 ml of extract was dissolved in alcohol solution and the mixture was added to 10 ml of distilled water | No precipitation was formed | - |
| 13. | Phenols | To 1 ml of extract, 2 ml of distilled water were added and followed by a few drops of 10% ferric chloride | Colour changed to green | + |
| 14. | Phlobatanins | To 1 ml of extract, 2% HCL was added and the mixture was heated to boiling | No precipitate was formed | - |
| 15. | Anthraquinones | To 1 ml of extract, 1 ml conc HCL was added followed by a few drops of 10% FeCl_3_ and diethylether. 1 ml of conc. Ammonium solution was also added | No observable change | - |

**Key: + = Present;**

**- = Absent**
